# The *In Vitro* and *In Vivo* Anti-Cancer Activities of a Standardized Quassinoids Composition from *Eurycoma longifolia* on LNCaP Human Prostate Cancer Cells

**DOI:** 10.1371/journal.pone.0121752

**Published:** 2015-03-31

**Authors:** Kind Leng Tong, Kit Lam Chan, Sazaly AbuBakar, Bin Seng Low, Hai Qiu Ma, Pooi Fong Wong

**Affiliations:** 1 Department of Pharmacology, Faculty of Medicine, University of Malaya, 50603 Kuala Lumpur, Malaysia; 2 School of Pharmaceutical Sciences, University of Science Malaysia, 11800 Minden, Penang, Malaysia; 3 Department of Medical Microbiology, Faculty of Medicine, University of Malaya, 50603 Kuala Lumpur, Malaysia; Indiana University, UNITED STATES

## Abstract

Quassinoids are a group of diterpenoids found in plants from the Simaroubaceae family. They are also the major bioactive compounds found in *Eurycoma longifolia* which is commonly used as traditional medicine in South East Asia to treat various ailments including sexual dysfunction and infertility. These uses are attributed to its ability to improve testosterone level in men. Chronic consumption of *E*. *longifolia* extracts has been reported to increase testosterone level in men and animal model but its effect on prostate growth remains unknown. Therefore, the present study investigates the effects of a standardized total quassinoids composition (SQ40) containing 40% of the total quassinoids found in *E*. *longifolia* on LNCaP human prostate cancer cell line. SQ40 inhibited LNCaP cell growth at IC_50_ value of 5.97 μg/mL while the IC_50_ on RWPE-1 human prostate normal cells was 59.26 μg/mL. SQ40 also inhibited 5α-dihydrotestosterone-stimulated growth in LNCaP cells dose-dependently. The inhibitory effect of SQ40 in anchorage-independent growth of LNCaP cells was also demonstrated using soft agar assay. SQ40 suppressed LNCaP cell growth via G_0_/G_1_ phase arrest which was accompanied by the down-regulation of CDK4, CDK2, Cyclin D1 and Cyclin D3 and up-regulation of p21^Waf1/Cip1^ protein levels. SQ40 at higher concentrations or longer treatment duration can cause G_2_M growth arrest leading to apoptotic cell death as demonstrated by the detection of poly(ADP-ribose) polymerase cleavage in LNCaP cells. Moreover, SQ40 also inhibited androgen receptor translocation to nucleus which is important for the transactivation of its target gene, prostate-specific antigen (PSA) and resulted in a significant reduction of PSA secretion after the treatment. In addition, intraperitoneal injection of 5 and 10 mg/kg of SQ40 also significantly suppressed the LNCaP tumor growth on mouse xenograft model. Results from the present study suggest that the standardized total quassinoids composition from *E*. *longifolia* promotes anti-prostate cancer activities in LNCaP human prostate cancer cells.

## Introduction

Quassinoids are a group of diterpenoids found in plants of the family of Simaroubaceae which possess bioactivities such as anti-tumor [1,2], anti-tuberculosis [3], anti-malarial [4,5], anti-ulcer [6,7], insect growth regulating [8], anti-HIV [9] and anti-inflammatory [10,11]. Their anti-cancer activity was extensively discussed in previous reviews [12,13]. Quassinoids were reported as the major components found in *Eurycoma longifolia* [14]. *E*. *longifolia* belongs to the plant family Simaroubaceae and is locally known as “Tongkat Ali” or “Pasak Bumi” in Malaysia and Indonesia, “Ian-Don” in Thailand and “Cay ba binh” in Vietnam [15]. *E*. *longifolia* is a popular herb used traditionally to improve male libido, sexual prowess and fertility. Due to its unique testosterone enhancing property, the crude extracts of this plant is now widely marketed and used to increase male virility and correct sexual dysfunction [14,15]. Several studies have shown that consumption of the extract increased production of testosterone and contributed to the improved sperm quality in men with idiopathic infertility and testosterone level of late-onset hypogonadism [16] and in androgen-deficient osteoporosis animal model [17]. The increased production of testosterone by *E*. *longifolia* has been attributed to the increase in human chorionic gonadotropin level [18] and the inhibition of the activity of phosphodiesterase and aromatase conversion of testosterone to oestrogen which subsequently triggers hypothalamic-pituitary-gonadal axis to increase testosterone levels [19,20].

Androgens such as testosterone and 5α-dihydrotestosterone (DHT) are important for the development, maturation, and function of the prostate gland. Nevertheless, deregulation of the androgen receptor (AR) pathway has been implicated in benign and malignant prostate disorders, such as benign prostatic hypertrophy (BPH) and prostate cancer [21,22]. Since elevation of testosterone has been associated with an increase in risk for prostate carcinogenesis [23], is mitogenic in prostatic cells [24–26] and has been shown to be a strong tumor promoter in rodent’s prostate [27], we undertook the present study to determine whether *E*. *longifolia* extract promotes or inhibits prostate cancer cell growth.

## Materials and Methods

### Ethics statement

Experiment with mice was performed in accordance to the protocol approved by the Faculty of Medicine Institutional Animal Care and Use Committee, University of Malaya (Ethics Reference Number: 2013-06-07/PHAR/WPF). The entire experiment was performed in the AAALAC International accredited Animal Experimental Unit of the Faculty of Medicine, University of Malaya.

### Preparation of a standardized quassinoids composition from *E*. *longifolia*

A standardized quassinoids composition (SQ40) containing 40% of the total quassinoids in *E*. *longifolia* was prepared according to the method of Low’s study [28]. Briefly, the air-dried powdered roots (15 kg) of the *E*. *longifolia* were extracted with 6 × 4 L of 95% methanol for 6 days at 60°C. The combined methanol extract upon evaporation to dryness under partial vacuum yielded a dark brown residue of 450 g (3% w/w), which was next chromatographed on a pre-packed Diaion HP 20 (Mitsubishi Chemical, Tokyo, Japan) resin column. The chosen quassinoid-rich fraction, SQ40 was derived by elution with a gradient of H_2_O-MeOH mixtures (1:0 to 0:1) at decreasing polarity [20], and subsequently dried under partial vacuum to 45 g (10% w/w of crude extract). The high performance liquid chromatographic (HPLC) analysis quantified the major quassinoids as 32.16% w/w in SQ40, comprising 14.49 ± 0.26% of eurycomanone, 7.39 ± 0.17% epoxyeurycomanone, 0.72 ± 0.06% 13,21-dihydroeurycomanone and 9.54 ± 0.22% w/w eurycomanol [19]. These quassinoids were isolated and their purified structures (> 95%) were identified and confirmed following the protocol described previously [29–31]. The purity of the compounds was determined with Empower 2 workstation software (Waters, Milford, MA, USA) operated in a Waters Delta Prep HPLC system equipped with a Waters 2996 photodiode array detector.

### Preparation of charcoal-stripped serum (CSS)

Charcoal-stripped serum (CSS) was prepared as described previously to deplete the growth factors present in the serum [32]. Briefly, 5 g of dextran-coated charcoal (Sigma-Aldrich, St. Louis, MO) was added to 500 mL of fetal bovine serum (FBS; Sigma-Aldrich, St. Louis, MO) and mixed gently for 1 hour. The FBS was then centrifuged at 2500 x g for 10 minutes under sterile condition. Serum supernatant was then collected and subjected to a second cycle of dextran-coated charcoal treatment. Finally, the serum was filtered through a 0.2 μm porous membrane (Orange Scientific, Braine-l'Alleud, Belgium) and stored at -20°C for further use. The sterility of CSS was checked by incubating portion of the media in a humidified atmosphere containing 5% CO_2_ for 14 days.

### Cell culture

Human prostate cancer, LNCaP and PC-3 cells, human normal prostate, RWPE-1 cells and human normal liver, WRL 68 cells were purchased from the American Type Culture Collection (ATCC, Manassas, USA). LNCaP cells were derived from supraclavicular lymph node of patient whose prostate cancer was exhibiting androgen independent growth. LNCaP cells are androgen sensitive and express prostate-specific antigen (PSA), prostatic acid phosphatase and AR [33]. LNCaP cells have a single point mutation of codon 868 (Thr to Ala) in androgen-binding domain of the AR and respond not only to androgens but also to antiandrogens, estrogens and progestins [34]. PC-3 cells were derived from bone metastases of a patient with grade IV prostatic adenocarcinoma and does not respond to androgens, glucocorticoids, or epidermal or fibroblast growth factors [35]. RWPE-1 cells were the non-neoplastic adult human prostatic epithelial cells from peripheral zone of a histologically normal adult human prostate and was immortalized with human papillomavirus 18 [36]. LNCaP and PC-3 cells were cultured in Roswell Park Memorial Institute Medium (RPMI 1640; Invitrogen, Carlsbad, USA) supplemented with 10% v/v FBS and 1% v/v penicillin-streptomycin (Invitrogen, Carlsbad, USA) while RWPE-1 cells were cultured in Keratinocyte-Serum Free Media (K-SFM; Invitrogen, Carlsbad, USA) supplemented with 0.5% v/v penicillin-streptomycin. WRL 68 cells were cultured in Dulbecco’s Modified Eagle Medium (DMEM; Invitrogen, Carlsbad, USA) supplemented with 10% v/v FBS and 1% v/v penicillin-streptomycin. All cell lines were maintained at 37°C in humidified atmosphere of 5% CO_2_ in air.

### Cell viability assay

The effect of SQ40 on the viability of LNCaP, PC-3, RWPE-1 and WRL 68 cells was determined by 3-[4,5-dimethylthiazol-2yl]-2,5-diphenyl tetrazoliumbromide (MTT; Invitrogen, Carlsbad, USA) assay. Briefly, the cells were plated on 96-well plates at optimal cell density of approximately 1.5 x 10^4^ LNCaP cells/well; 1.25 x 10^4^ PC-3 cells/well; 1.5 x 10^4^ RWPE-1 cells/well; 1.25 x 10^4^ WRL 68 cells/well. After 24 hours, the cells were treated with increasing concentrations (2.5–100 μg/mL) of SQ40 for 72 hours. At the end of incubation, MTT assay was performed as described previously [37]. The cell viability was calculated as percentage of cell viability compared to vehicle control cells, assigned as 100% viability. Finally, the half maximal inhibitory concentration (IC_50_) was determined using GraphPad Prism software version 5.0 (GraphPad Software Inc., San Diego, CA).

### Dihydrotestosterone (DHT) treatment

LNCaP cells treated with 5α-androstan-17β-ol-3-one (DHT; Sigma-Aldrich, St. Louis, MO) were used to investigate the responsiveness of these cells to mitogenic stimulation by DHT. Prior to the treatment, LNCaP cells were grown in RPMI supplemented with 5% CSS for 48 hours to prevent interference of androgens and other growth factors present in complete FBS. Approximately 1.5 x 10^4^ LNCaP cells/well were seeded onto 96-well plate and treated with increasing concentrations (20–140 nM) of DHT with or without 3, 6 and 12 μg/mL of SQ40 for 72 hours. Cell viability was determined by MTT assay.

### Soft agar colony formation assay

LNCaP cells were pre-treated with SQ40 at its IC_50_ value for 72 hours. Prior to seeding onto soft agar, single cell suspension was obtained by passing the cells through a fine needle. Five thousand SQ40-treated and vehicle-controlled cells were mixed with growth media containing 0.3% agar and seeded on top of a base layer containing 0.5% agar in growth media in 60 mm petri dishes. The cultures were incubated at 37°C in humidified atmosphere of 5% CO_2_ in air for another 3 weeks. The media were replenished every 3 days with fresh growth medium. At the end point of the experiment, only colonies larger than 0.5 mm were counted under the microscope.

### Real-time cell proliferation analysis

The growth kinetics of LNCaP and RWPE-1 cells were examined real-time by Real-Time Cell Analysis (RTCA) System (Roche Diagnostics, Mannheim, Germany) as previously described [37]. The impedance will increase when adherent cells attach and spread across the sensor surface of an electrode and decrease when the cells round up and detach. Briefly, 50 μL of completed culture medium was added to each well of E-plate 16 and background reading was recorded. A cell suspension of 50 μL at cell density of 3.0 x 10^4^ cells/well was then added into each well of E-plate 16. The changes in impedance due to cell attachment, proliferation and spreading were monitored overnight. When the cells entered logarithmic growth phase, the cells were treated with 2.5–80 μg/mL of SQ40. The cells treated with complete growth medium were referred as vehicle control while 5 μM paclitaxel-treated cells were referred as positive control. The cells were then monitored for another 72 hours. The impedance values were expressed as the Cell Index (CI). The growth curves were normalized to the CI of the last measured time point before the addition of drugs or vehicle control.

### Trypan blue exclusion test

Confluent LNCaP cells were treated with vehicle culture medium, 3, 6, 12 and 20 μg/mL of SQ40 for 72 and 96 hours. LNCaP cells treated with 1 μM paclitaxel for 72 and 96 hours were used as positive control and the untreated cells were used as negative control, respectively. The treated and vehicle control cells were harvested at the end point of incubation period and the cells suspensions were mixed with 0.4% trypan blue solution (Sigma-Aldrich, St. Louis, MO) at a ratio of 1:1 and incubated for 3–5 minutes. The stained and unstained cells were counted using a hemocytometer chamber. Percentage of dead cells at each concentration was calculated according to the equation below.

$$\frac{\text{number of stained cells}}{\text{number of stained}+\text{unstained cells}}\times 100\text{\%}=\text{Percentage of dead cells}$$ (1)

### Cell cycle analysis

Confluent LNCaP cells were treated with vehicle culture medium, 3, 6 and 12 μg/mL of SQ40 for 24, 48 and 72 hours. Cell cycle analysis was performed using Cycle TEST PLUS DNA Reagent Kit (BD Biosciences, New Jersey, USA). At the end of the incubation period, the treated cells were harvested, washed and stained with propidium iodide according to the manufacturer’s instruction. After staining, the samples were analyzed by FACSCanto II flow cytometry (BD Biosciences, New Jersey, USA). A minimum of 20000 events per sample were recorded for each sample and the data was analyzed using Flow Cytometry DNA Modeling Software, ModFit LT 3.2 (Verity Software House, USA).

### Immunoblot Analysis

The protein expression of G_1_/S regulators such as cyclin-dependent kinase (CDK), CDK4, CDK2, Cyclin D1, Cyclin D3, p21^Waf1/Cip1^ and p27^Kip1^ and cleaved-poly(ADP-ribose) polymerase (PARP) were analyzed by immunoblotting. Cell lysates were prepared using radioimmunoprecipitation assay (RIPA) Lysis Buffer System in the presence of 1% phenylmethylsulfonyl fluoride (PMSF) solution, 1% sodium orthovanadate solution and 1% protease inhibitor cocktail solution. Protein concentration of the cell lysates were determined by Pierce BCA Protein Assay Kit (Thermo Scientific, Walthan, MA). Equal amount of proteins were subjected to 12% sodium docecyl sulphate-polyacrylamide gel electrophoresis (SDS-PAGE) and then transferred onto polyvinylidene fluoride (PVDF) membrane (pore size 0.45 μm; Milipore, Bedford, MA). The membranes were then probed with primary antibodies against CDK4, CDK2, Cyclin D1, Cyclin D3, p21^Waf1/Cip1^, p27^Kip1^ and cleaved-PARP (Asp214) (D64E10) (Cell Signaling Technology, Danvers, MA). This step was followed by incubation with the appropriate secondary antibodies conjugated to horseradish peroxidase. After the washing steps, the protein bands were visualized colorimetrically using 3,3,5,5 tetramethylbenzine (TMB; Sigma Aldrich, Louis, MO) solution and quantified using a gel documentation system (BioRad, Richmond, Calif).

### Androgen receptor (AR) and Prostate specific antigen (PSA) ELISA

Briefly LNCaP cells were cultured in RPMI 1640 supplemented with 5% CSS for 48 hours. The cells were then treated with 3, 6 and 12 μg/mL of SQ40 with or without 100 nM DHT for another 72 hours. DHT was added to stimulate AR translocation. The nuclear fraction was obtained from the cell lysate using Nuclear Extraction Kit (Cayman Chemical, MI, USA) and the nuclear AR level was measured using Nuclear Receptor Sandwich AR ELISA (Active Motif, Carlsbad, USA). AR level was normalized to the total nuclear protein level. In the same experiment, growth medium of the vehicle control and treated cells was collected and the secreted PSA level was quantified by Prostate Specific Antigen Human ELISA Kit (Abcam, MA, USA). The PSA values were normalized against total cell number.

### *In vivo* LNCaP xenograft study

Male NCr immunodeficient mice, 5 weeks old, 20–25 grams were purchased from InVivos Pte. Ltd. (Singapore) and housed in individually ventilated cages under specific pathogen-free conditions and maintained in a 12 hour light-dark cycle at 24°C. The animals were provided with sterile food and water ad libitium. The mice were anaesthetized prior the injection of LNCaP cells (2 x 10^6^) with 50% matrigel (0.1 mL; Becton Dickinson, Jersey, USA) into the right flank of the nude mouse subcutaneously. Treatment was initiated when the tumors were palpable. Tumor-bearing mice were randomly assigned to 4 groups. Each group consisted of 6 animals. These groups of mice were then given intraperitoneal injections of vehicle solution (saline), 5 mg/kg of paclitaxel (positive control), 5 and 10 mg/kg SQ40 thrice a week for 6 weeks. Tumor size was palpated and the length and width were measured with a caliper. The tumor volume was calculated using the formula, 0.5 x length x width^2^. The weight and tumor volume of each mouse were measured once a week over a period of 6 weeks. The mice were sacrificed once the tumor nodules reached 1.5 cm in diameter.

### Statistical analysis

All assays were performed in at least three separate experiments. The data were presented as mean ± standard error of the mean (SEM). Statistical analyses were performed using one-way analysis of variance (ANOVA), with Bonferroni’s Multiple Comparison Test using the GraphPad Prism software (version 5.0). Statistical significance was expressed as *, *p*<0.05; **, *p*<0.01; ***, *p*<0.001 versus vehicle control and ^**#**^, *p*<0.05; ^**##**^, *p*<0.01; ^**###**^, *p*<0.001 versus 100 nM DHT-stimulated cells.

## Results

### SQ40 is selectively cytotoxic to LNCaP prostate cancer cells and inhibited DHT-stimulated growth of LNCaP cells

**Fig 1:**
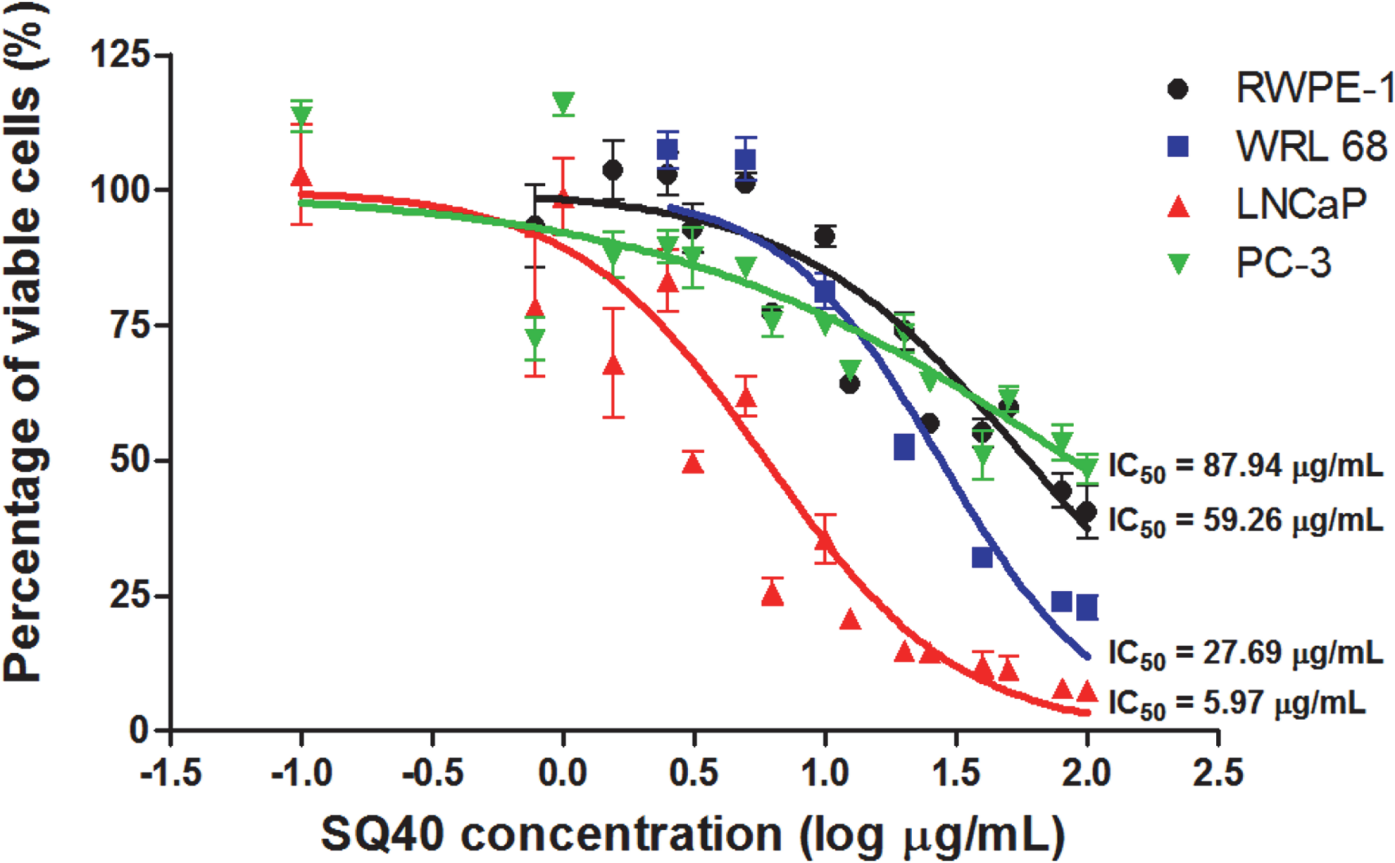
Dose-dependent cytotoxicity of SQ40 on human normal and prostate cancer cell lines. RWPE-1 normal prostate cells (black), WRL 68 normal liver cells (blue), LNCaP (red) and PC-3 prostate cancer cells (green) were treated with increasing concentrations (2.5–100 μg/mL) of SQ40 for 72 hours and cell viability was measured using MTT reduction assay. Data were expressed as mean ± SEM of four independent experiments. IC_50_ values of SQ40 on RWPE-1, WRL 68, LNCaP and PC-3 cells at 72 hours treatment were 59.26 μg/mL, 27.69 μg/mL, 5.97 μg/mL and 87.94 μg/mL, respectively.

*In vitro* cytotoxicity activity of SQ40 was examined on human normal prostate, RWPE-1 cells, human normal liver, WRL 68 cells, human prostate cancer, PC-3 and LNCaP cells. The concentrations that caused half maximal inhibitory effect (IC_50_) on RWPE-1 and WRL 68 cells were 59.26 μg/mL and 27.69 μg/mL, respectively (Fig. 1, black and blue line). SQ40 inhibited LNCaP cell growth at IC_50_ of 5.97 μg/mL in a dose-dependent manner (Fig. 1, red line). The IC_50_ values of the quassinoids composition on RWPE-1 and WRL-68 cells were higher compared to that of LNCaP cells, suggesting that SQ40 was more selective to LNCaP cancer cells than the normal cells. On the other hand, SQ40 inhibited 50% of cell growth on PC-3 cells at concentration higher than (87.94 μg/mL; Fig. 1, green line) that of LNCaP cells suggesting that the quassinoid-rich fraction affected the LNCaP cells but not the PC-3 prostate cancer cells.

**Fig 2:**
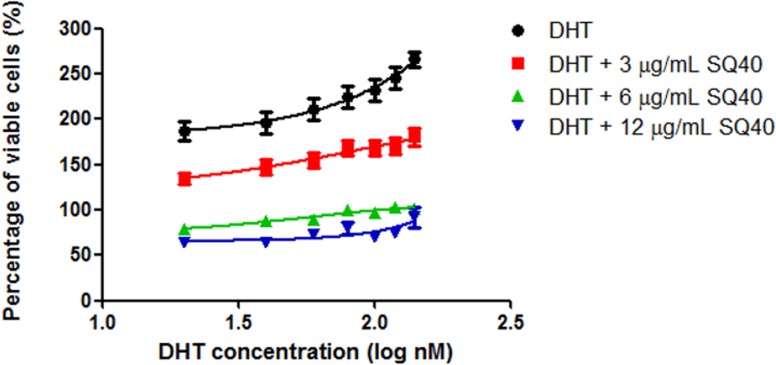
The effect of SQ40 on cell viability of DHT-stimulated LNCaP cells. LNCaP cells were treated with increasing concentrations of DHT with or without 3, 6 and 12 μg/mL of SQ40 for 72 hours in RPMI 1640 supplemented with 5% CSS and cell viability was measured using MTT reduction assay. Percentage of viable cells was calculated relative to vehicle control cells without DHT treatment. Data were expressed as mean ± SEM of three independent experiments.

The cytotoxic effect of SQ40 was further evaluated on androgen-stimulated LNCaP prostate cancer cells. LNCaP cells were first allowed to grow in growth medium which supplemented with 5% CSS and DHT was added to stimulate cell proliferation. In the presence of increasing concentrations of DHT, LNCaP cells were stimulated to grow exponentially. Co-treatment with 3, 6 and 12 μg/ml SQ40 inhibited LNCaP cell growth by more than 50% compared to cells stimulated with DHT alone (Fig. 2).

### SQ40 suppressed anchorage-independent growth of LNCaP cells

**Fig 3:**
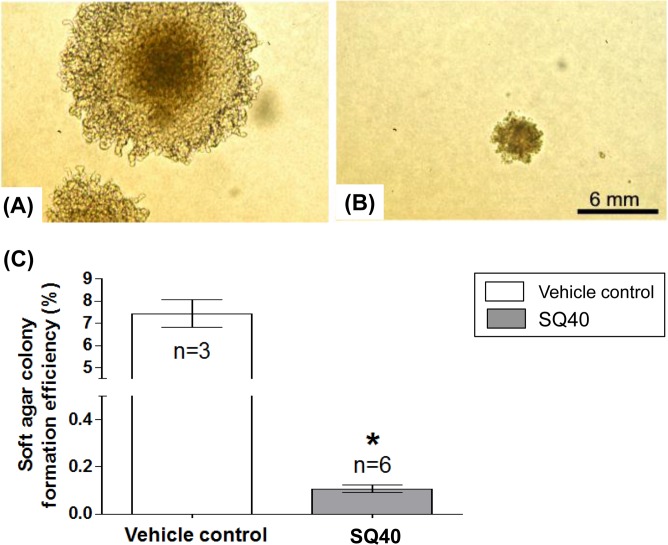
The effects of SQ40 on LNCaP cells anchorage-independent growth. LNCaP cells were pre-treated with SQ40 or vehicle control for 72 hours and then plated on the soft agar media for another 3 weeks. Representative colonies of (**A**) vehicle control and (**B**) SQ40-treated LNCaP cells are shown. (**C**) Graphical representations of soft agar colony formation efficiency. Data were expressed as mean ± SEM of three independent experiments for vehicle control cells and six independent experiments for SQ40-treated cells. * indicates *p*<0.05 versus vehicle control.

The effect of SQ40 on LNCaP cells anchorage-independent growth was investigated using soft agar assay. The LNCaP cells were pre-treated with SQ40 at its IC_50_ value for 3 days prior to plating on the soft agar media at an equal cell number with the vehicle control cells. The size of quassinoids composition-treated cell colonies was markedly reduced compared to the vehicle control cell colonies (Fig. 3A and 3B). In addition, the colony formation efficiency of SQ40-treated LNCaP cells was significantly reduced when compared to the vehicle control (Fig. 3C; *p*<0.05). This finding suggests that SQ40 inhibited anchorage-independent growth of LNCaP prostate cancer cells.

### SQ40 inhibited LNCaP cell growth at low concentration but induced cell death at high concentration

**Fig 4:**
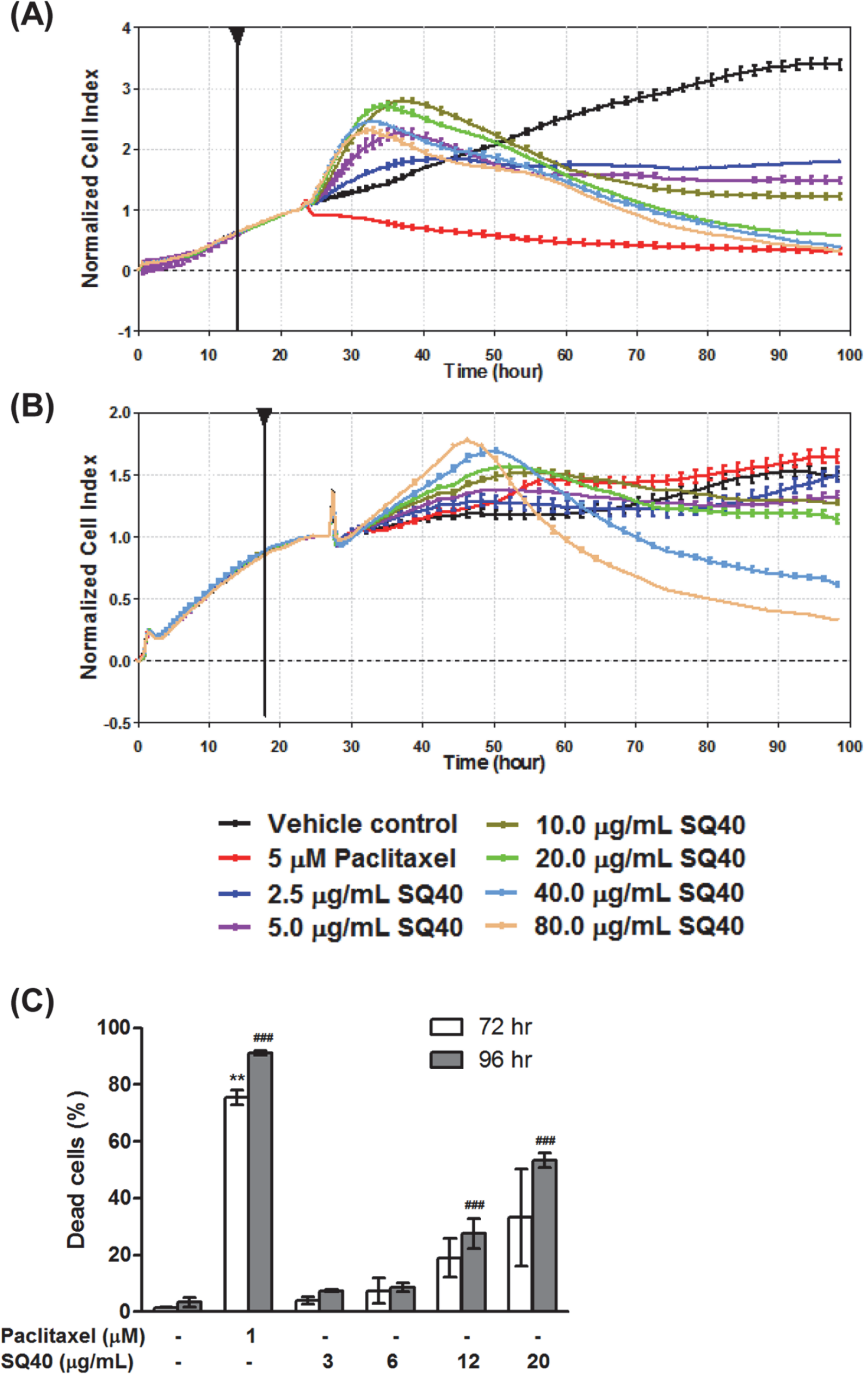
Growth profile of LNCaP and RWPE-1 cells upon treatment with SQ40. The growth kinetics of (**A**) LNCaP and (**B**) RWPE-1 cells were examined real-time using RTCA. The impedance values were recorded in real-time and were expressed as the Cell Index (CI). Cells treated with growth media alone were referred as vehicle control while 5 μM paclitaxel-treated cells were referred as positive control. (**C**) SQ40-treated LNCaP cells were stained with 0.4% trypan blue solutions in a ratio of 1:1 after 72 and 96 hours of treatment respectively. Cells treated with growth media alone were referred as vehicle control while 1 μM paclitaxel-treated cells were referred as positive control. Data were expressed as means ± SEM of three independent experiments. ** indicates *p*<0.01 versus 72-hour treated vehicle control. ^##^ indicates *p*<0.01; ^###^, *p*<0.001 versus 96-hour treated vehicle control.

Growth kinetics of LNCaP and RWPE-1 cells were monitored in real-time using an impedance-based cell sensing measurement system. It was observed that the normalized CI values for SQ40-treated LNCaP cells showed a steady decline after 6 hours of treatment with the quassinoids composition. After 72 hours of treatment, it was observed that the LNCaP cells treated with lower concentrations (2.5–10 μg/mL) of SQ40 only scored about half of the normalized CI values of vehicle control cells and this suggests that SQ40 at low concentrations exerts cytostatic effect on LNCaP cells. However, SQ40 at higher concentrations (20–80 μg/mL) resulted in very low normalized CI values similar to that of the positive control, 5 μM paclitaxel-treated cells (Fig. 4A).

In contrast, the normal prostate RWPE-1 cells treated with < 20 μg/mL of SQ40 showed normalized CI values that were similar to those of the vehicle control cells suggesting that SQ40 at these doses did not induce cytotoxic effect on normal prostate cells. However, cytotoxicity was observed on RWPE-1 cells at very high concentrations of the quassinoids composition (40 and 80 μg/mL; Fig. 4B). In all subsequent studies, 3, 6, and 12 μg/mL of SQ40 were used to treat LNCaP cells. To demonstrate that subsequent molecular effects of SQ40 at these doses were not contributed by the high percentage of dead cells, trypan blue exclusion staining was performed to determine the percentage of viable cells in the cultures. After 72 hours of treatment, the percentage of dead cells were ~20% and 35% in 12 and 20 μg/mL SQ40-treated LNCaP cells when compared to the untreated control. An extended treatment duration of 96 hours increased the percentage of dead cells to ~30% and ~60% in 12 and 20 μg/mL SQ40-treated LNCaP cells, respectively (Fig. 4C; *p*<0.001).

### SQ40 induced G_0_/G_1_ phase arrest in LNCaP cells

**Fig 5:**
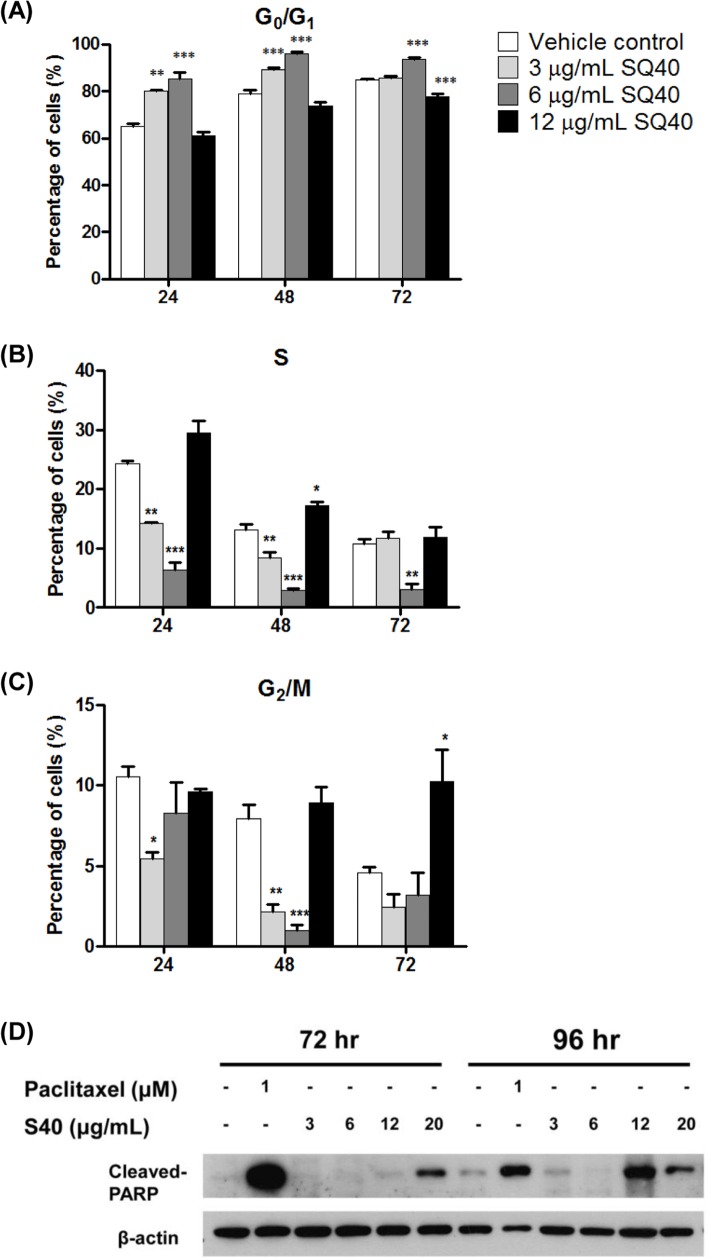
Cell cycle distribution of LNCaP cells upon SQ40 treatment. LNCaP cells were treated with growth media (vehicle control), 3, 6 and 12 μg/mL of SQ40. Cell distribution in (**A**) G_0_/G_1_, (**B**) S and (**C**) G_2_/M phase at 24, 48 and 72 hours treatment were analysed by FACSCanto II flow cytometry and evaluated using ModFit cell cycle analysis software. Data were expressed as means ± SEM of three independent experiments. * indicates *p*<0.05; **, *p*<0.01; ***, *p*<0.001 versus vehicle control. (**D**) Protein expression of cleaved-PARP in LNCaP cells upon SQ40 treatment for 72 and 96 hours. β-actin served as a loading control.

Cell cycle analysis was performed to investigate the inhibitory effect of SQ40 in cell cycle progression of LNCaP cells. LNCaP cells at 70–80% confluent were treated with 3, 6 and 12 μg/mL of the quassinoids composition for 24, 48 and 72 hours. As shown in Fig. 5, SQ40 significantly arrested LNCaP cells at G_0_/G_1_ phase in a dose- and time-dependent manner. At 24-hour treatment, LNCaP cells showed higher G_0_/G1 phase cell population at 80.22% (*p*<0.01) in 3 μg/mL quassinoids composition-treated cells and 85.45% (*p*<0.001) in 6 μg/mL quassinoids composition-treated cells compared to the vehicle control cells (65.22%; Fig. 5A). The cell population of 6 μg/mL quassinoids composition-treated LNCaP cells in G_0_/G_1_ phase significantly increased to 96.15% (*p*<0.001) and 93.67% (*p*<0.001) after 48- and 72-hour treatment, respectively (Fig. 5A). The increase in the percentage of cell population in G_0_/G_1_ phase was accompanied by a decrease in the percentage of cell population in S phase (3.11%; *p*<0.01) and G_2_/M phase (3.21%) on LNCaP cells after 72-hour treatment (Fig. 5D). These significant changes suggest that SQ40 inhibited the growth of LNCaP cells by arresting the cells at G_0_/G_1_ phase.

On the other hand, LNCaP cells treated with 12 μg/mL of SQ40 showed a significant increase in G_2_/M phase after 72 hours of treatment. The G_2_/M phase cell population in 12 μg/mL SQ40-treated LNCaP cells was 9.58%, 8.94% and 10.23% (*p*<0.01) after 24-, 48-, and 72- hour treatments, respectively, whilst those of the vehicle control cells were 10.55%, 7.95% and 4.58% after 24-, 48-, and 72-hour treatment, respectively (Fig. 5C). In addition, cleaved-PARP, an apoptotic marker was detected when the treatment duration with 12 μg/mL of SQ40 was extended to 96 hours. Moreover, cleaved-PARP was also significantly detected at 72 hours when the concentration of SQ40 was increased to 20 μg/mL (Fig. 5D). Taken together, these findings suggest that SQ40 induced cell death following G_2_/M arrest.

### SQ40 inhibited the growth of LNCaP cells by down-regulating CDK4, CDK2 and Cyclin D1 proteins and up-regulating p21^WAf1/Kip1^ protein expression level

**Fig 6:**
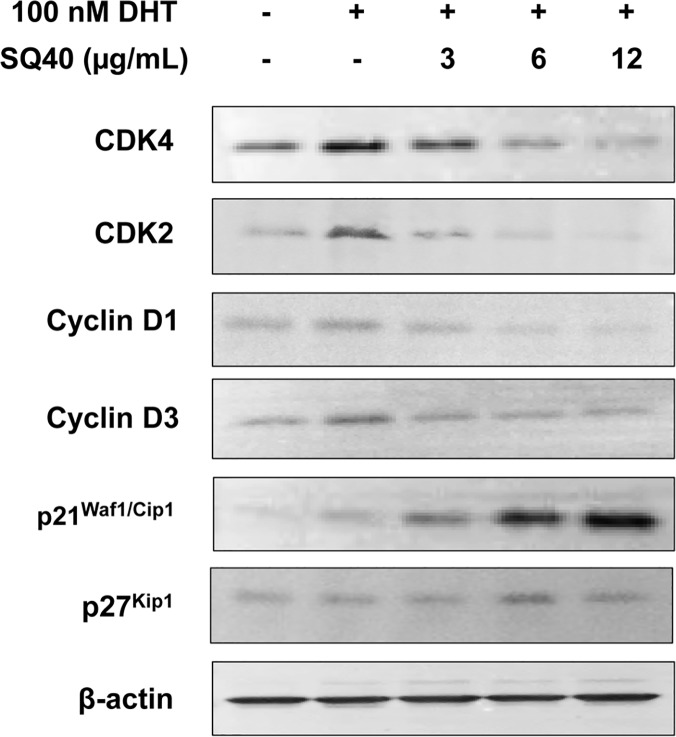
Protein expression of G_1_/S regulatory proteins in LNCaP cells treated with SQ40. LNCaP cells were first cultured in growth media supplemented with 5% CSS for 48 hours and then treated with 3, 6 and 12 μg/mL of SQ40 with the presence of 100 nM DHT for 72 hours. Immunoblotting was performed on protein extracts to detect CDK4, CDK2, Cyclin D1, Cyclin D3, p21^Waf1/Cip1^ and p27^Kip1^. β-actin served as a loading control.

Immunoblot analysis was performed to investigate the effects of SQ40 on cell cycle regulatory protein expression. The expression level of G_1_/S regulatory protein including CDK4, CDK2, Cyclin D1 and Cyclin D3 were up-regulated in LNCaP cells when stimulated to proliferate with DHT, a metabolite of testosterone (Fig. 6). The presence of SQ40 in 100 nM DHT-stimulated LNCaP cells down-regulated the protein expression levels of CDK4, CDK2, Cyclin D1 and Cyclin D3 in a dose-dependent manner (Fig. 6). In addition, SQ40 increased the protein expression level of cell cycle inhibitor p21^Waf1/Kip1^ in the presence of 100 nM DHT, however, the quassinoids composition did not affect the expression level of p27^Kip1^ after 72 hours of treatment in LNCaP cells. These results further supported the flow cytometry findings that SQ40 arrested cells at G_0_/G_1_ phase.

### SQ40 suppressed AR protein level and decreased the production of PSA in LNCaP cells

**Fig 7:**
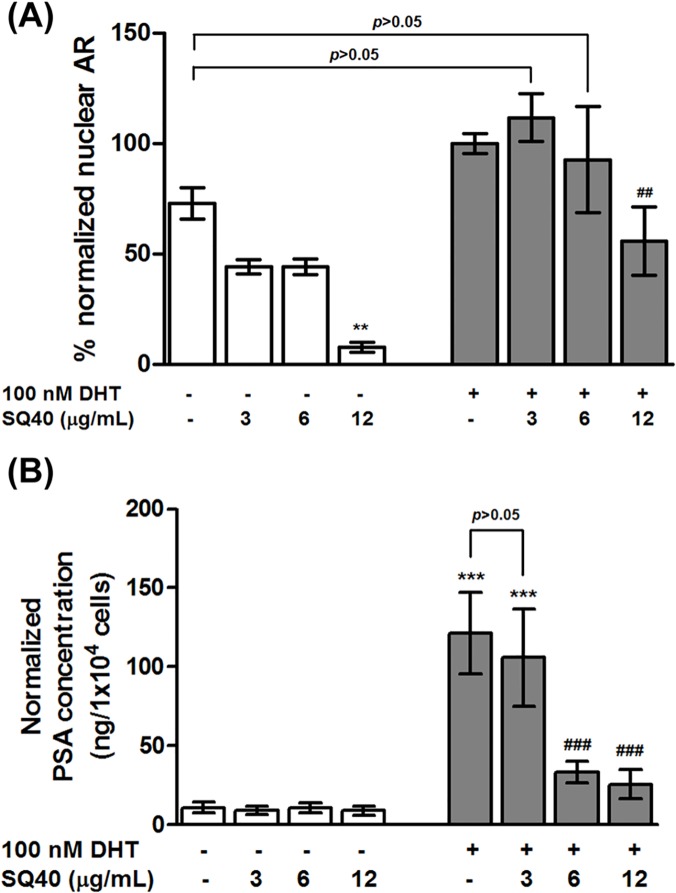
Quantitative measurement of the level of nuclear AR and PSA secretion in SQ40-treated LNCaP cells. LNCaP cells were first cultured in growth media supplemented with 5% CSS for 48 hours and then treated with 3, 6 and 12 μg/mL of SQ40 in the presence or absence of 100 nM DHT for 72 hours. Nuclear fraction of AR obtained from the cell lysate and concentrations of PSA secreted into culture medium were measured using commercial available ELISA kit. (**A**) AR level was normalized to the total nuclear protein level while (**B**) the concentration of PSA were normalised to the total cell number. Data were expressed as means ± SEM of three independent experiments and indicated as percentage of 100 nM DHT-stimulated cells set at 100%. ** indicates *p*<0.01; ***, *p*<0.001 versus unstimulated control. ^**###**^, *p*<0.001 versus 100 nM DHT-stimulated cells.

The effect of SQ40 on the AR protein of LNCaP was investigated next. Nuclear AR extract in 100 nM DHT stimulated-LNCaP cells increased by 25% compared to the unstimulated cells (Fig. 7A). SQ40 treatment at 3 and 6 μg/mL alone decreased the basal nuclear AR level by 25% as well when compared to the unstimulated cells (Fig. 7A; *p*>0.05). Therefore, combination treatment of DHT and the quassinoids composition at 3 and 6 μg/mL did not significantly affect the nuclear AR level when compared to the unstimulated cells (Fig. 7A; *p*>0.05). However, treatment with 12 μg/mL of SQ40 significantly suppressed the basal nuclear AR level and its suppression could not be overcome by the presence of DHT (Fig. 7A; *p*<0.001).

The translocation of activated AR induces the transcription of androgen-responsive gene, such as PSA. Therefore, the level of secreted PSA was measured using ELISA to assess whether AR-mediated functional responses would be affected by SQ40 treatment. PSA secretion increased significantly by ~100% in cells upon stimulation with 100 nM DHT alone compared to the unstimulated cells (Fig. 7B; *p*<0.001). The elevation of PSA secretion was significantly suppressed by 6 μg/mL (Fig. 7B; *p*<0.001) and 12 μg/mL SQ40 (Fig. 7B; *p*<0.001) but no significant change was observed when the cells were treated with 3 μg/mL SQ40 (Fig. 7B; *p*>0.05) when compared to DHT-stimulated cells. These results suggest that SQ40 inhibited AR translocation and this has led to the reduction of secreted PSA level.

### SQ40 inhibited LNCaP tumor xenograft growth in nude mice

**Fig 8:**
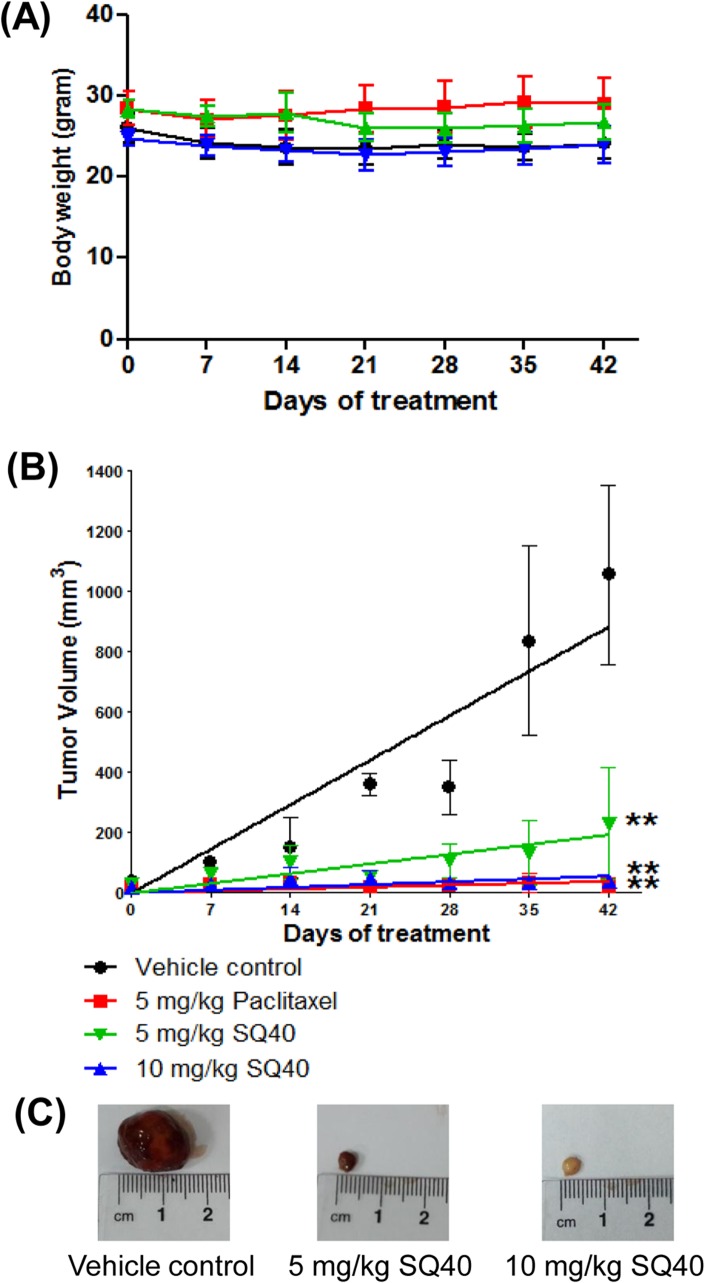
Anti-tumor activity of SQ40 against subcutaneous LNCaP cell tumors. LNCaP cells at 2 x 10^6^ were injected subcutaneously into right flank of NCr nude mice. SQ40 treatment was initiated when the tumor was palpable. Vehicle control (saline) and SQ40 were given intraperitoneally thrice a week for 6 weeks with a total of 18 doses. Graph of (**A**) mean body weight and (**B**) tumor volume for each treatment versus the number of days after initial injection of LNCaP cells. (**C**) Representative images of tumors isolated from vehicle control, 5 mg/kg and 10 mg/kg of SQ40-treated animals. Each point represents the mean ± SEM of data (n = 6). ** indicates *p*<0.01 versus vehicle control.

Results from the previous sections have demonstrated the *in vitro* anti-proliferative activities of quassinoid-rich fraction against prostate cancer cells. We next used LNCaP xenograft model to further investigate if SQ40 can suppress prostate tumor growth *in vivo*. LNCaP prostate cancer cells were inoculated subcutaneously onto NCr immunodeficient mice. Treatments with the vehicle control solution, 5 mg/kg of paclitaxel, 5 and 10 mg/kg of SQ40 were initiated when the tumors were palpable. All treatments were given intraperitoneally thrice a week for 6 weeks. The body weights of mice from vehicle control, paclitaxel-treated and quassinoids composition-treated animal groups were recorded from a range of 25 to 30 grams. The body weights of mice treated with 5 and 10 mg/kg of SQ40 did not significantly change when compared to that of vehicle control animal group (Fig. 8A). Treatment of 10 mg/kg of the quassinoids composition was shown as efficient as 5 mg/kg of paclitaxel, the standard prostate cancer chemotherapeutic drug in the suppression of LNCaP tumor growth. Intraperitoneal injection of 5 and 10 mg/kg of SQ40 significantly suppressed tumor growth 3 weeks after the administration of the treatment compared to the vehicle control mice (Fig. 8B). In addition, the tumor volume in 5 and 10 mg/kg quassinoids composition-treated animal group was significantly reduced by 4.6 fold (231.8 ± 185.6 mm^3^; *p*<0.01) and 58.3 fold (18.1 ± 11.2 mm^3^; *p*<0.01), respectively after 6 weeks of treatment compared to vehicle control mice (1054.9 ± 296 mm^3^; Fig. 8B). These results suggest that SQ40 also suppressed prostate tumor growth *in vivo*.

## Discussion

Earlier studies have shown that *E*. *longifolia* extract increased spermatogenesis by increasing testosterone levels [17,19,20,38]. Although, many previous reports showed that selected fractions of *E*. *longifolia* extract inhibit cell proliferation in different human cancer cell lines such as breast cancer, MCF-7 cells [39], liver cancer, HepG2 cells [40], cervical carcinoma, Hela cells [41], lung cancer, A549 cells [42] and leukemic, K-562 cells [43], its effect on the growth of prostate cancer cells remained unclear and is further compounded by the fact that increased testosterone level in plasma has been associated with an increase risk of prostate carcinogenesis. The present study was undertaken to determine whether the standardized quassinoids composition (SQ40) from *E*. *longifolia* stimulates or inhibits prostate cancer cell growth *in vitro* and *in vivo* models.

*In vitro* selective cytotoxic activity of SQ40 was shown in the present study on LNCaP human prostate cancer cells with IC_50_ value of 5.97 μg/mL compared to human normal prostate (59.26 μg/mL) and liver cells (27.69 μg/mL). SQ40 also reduced the colony formation efficiency and colony size of LNCaP cells and this indicates that its suppressive actions are extended to the non-adherent conditions of LNCaP cells. In normal prostate cells, no significant cytotoxicity was observed at concentrations (2.5–20 μg/mL) that inhibited LNCaP prostate cancer cells as shown by the real-time cell growth monitoring. WRL 68 human normal liver cells were used to compare the activities of SQ40. Liver is the vital organ in detoxification. The quassinoids composition was shown to be less cytotoxic to the normal liver cells, suggesting that the liver cells can metabolize the extracts. Although the IC_50_ for LNCaP was only ~4 times lower than the IC_50_ for normal liver cells, while the IC_50_ for PC-3 is significantly higher than both liver and prostate normal cells, the anti-cancer efficiency of SQ40 was substantiated in LNCaP xenograft in nude mice, where 10 mg/kg dose of SQ40 yielded similar efficacy as 5 mg/kg paclitaxel by suppressing the tumor volume for > 50-fold. These results suggest the selective cytotoxic activity of SQ40 on LNCaP prostate cancer cells.

Although p53 and caspase-9-mediated pathways were previously implicated in the cytotoxicity of *E*. *longifolia* fractions on MCF-7 and HepG2 cells [40,44], results from the present study showed that SQ40 inhibited LNCaP cell growth by arresting G_0_/G_1_ phase in a dose- and time-dependent manner. The cell growth inhibition activity of the quassinoids composition began as early as 6–8 hours after the addition of the quassinoids composition and at higher concentrations, the quassinoids composition is capable of inducing G_2_/M cell cycle arrest leading to cell death. This was confirmed by the detection of cleaved-PARP protein fragments in 12 and 20 μg/mL SQ40-treated LNCaP cell after 72 and 96 hours treatment. PARP is a 116kDa nuclear protein and functions in DNA damage. PARP cleavage is one of the hallmarks of apoptosis [45,46].

DHT, a metabolite of testosterone, stimulates cell cycle transition by up-regulating CDK4, CDK2 and their associated cyclin proteins. Active cyclin D-CDK4 complex phosphorylates and inhibits members of retinoblastoma (Rb) protein family including Rb1 and regulates cell cycle during G_1_/S transition [47]. Phosphorylated Rb1 allows the dissociation of transcription factor E2F from Rb/E2F complex and results in the transcription of various genes such as cyclin E, cyclin A, DNA polymerase, thymidine kinase and others, which are responsible for progression through G_1_ phase [48]. Cyclin E then forms a complex by binding to CDK2 and allows G_1_-to-S transition [49]. In the present study, the SQ40-induced cell cycle arrest at G_0_/G_1_ phase was due to its ability to suppress the DHT-induced protein expression of CDK4, CDK2 and Cyclin D1 in LNCaP. In addition, up-regulation of p21^Waf1/Cip1^ protein expression level was observed in SQ40-treated LNCaP cells. Protein p21 from cip/kip family binds and inhibits the activity of cyclin D-CDK4 or cyclin E–CDK2 complexes [50]. Moreover, p21, a downstream protein of p53 was previously reported to be up-regulated by eurycomanone in *E*. *longifolia* [40]. However, the quassinoids composition did not affect p27^Kip1^, another protein inhibitor of cyclin E and CDK2 by 72-hour of the quassinoids composition treatment on LNCaP cells. These data suggested that SQ40 inhibits G_1_-to-S transition by arresting G_0_/G_1_ phase via up-regulating expression of p21^Waf1/Kip1^ but not p27^Kip1^.

The binding of androgens such as testosterone or its metabolite, DHT activates AR translocation into nucleus and induces the transcription of androgen-responsive genes, which then regulate the key cellular processes including proliferation, differentiation, survival and apoptosis of prostate cells [51,52]. Our observation of significant reduction of nuclear AR and secreted PSA level in 12 μg/mL quassinoids composition-treated LNCaP cells in the presence of DHT stimulation suggests that SQ40 may exert growth inhibition in parts through the inhibition of the activities of AR protein in LNCaP prostate cancer cells. *E*. *longifolia* has been previously reported to have anti-estrogenic activity [31] and the present study further showed that it can also affect the AR protein of the LNCaP cells leading to cessation of growth.

The potential toxicity of SQ40 has been previously investigated. The oral median lethal dose (LD_50_) of a similar quassinoid-rich *E*. *longifolia* extract for female and male Sprague-Dawley rats was 1293 and > 2000 mg/kg, respectively [53]. In the same study, normal prostate histology with the absence of hyperplasia was also observed in tissues of rat fed chronically with the extract. Based on the reproductive toxicity and teratology studies, the no-observed adverse effect level (NOAEL) obtained in rats was 100 mg/kg body weight/day. It is recommended that any human dose derived from converting 100 mg/kg rat dose and below can be safely used for further clinical studies [53]. Li et al., 2013 also reported that *E*. *longifolia* extract is non-genotoxic and found no test compound related toxicity even after after 13-week consecutive exposure. They suggested an acceptable daily intake of 1.2 g/adult/day [54]. Results from this study on the inhibition of LNCaP prostate cancer cell growth together with the previous report of normal prostate histology of rats fed chronically with the extract [53], hence, alleviate the concern of the negative effects of testosterone increase induced by the extract on prostate cells.

## Conclusion

**Fig 9:**
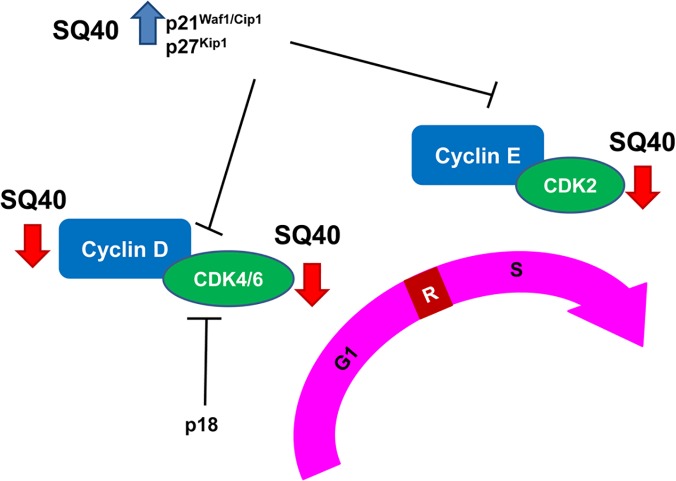
A schematic representation describing the anti-proliferation activities of SQ40 in regulation of cell cycle proteins in LNCaP cells.

Results from the present study showed that SQ40 induced selective cytotoxicity on human prostate cancer cells and inhibited the growth of LNCaP cells. SQ40 was shown to down-regulate the expression levels of G_1_-to-S phase transition regulatory proteins, Cyclin D1, CDK4 and CDK2 and up-regulate Cyclin inhibitor protein, p21^Waf1/Cip1^ which subsequently led to cell cycle arrest in G_0_/G_1_ phase (Fig. 9). Growth inhibition induced by SQ40 is associated with the inhibition of AR activities in parts as shown by the suppression of AR translocation into the nucleus from cytoplasm and reduction of the secretion of PSA by treatment of SQ40 in the presence of DHT stimulation. In addition, the anti-tumorigenic activity of SQ40 was successfully demonstrated in mouse xenograft model.
